# Organization of Community Psychiatric Services in Finland

**DOI:** 10.17816/CP64

**Published:** 2021-03-20

**Authors:** Jyrki Korkeila

**Affiliations:** University of Turku; Harjavalta Hospital

**Keywords:** community psychiatry, development, outcome, use of services, внебольничная психиатрия, развитие, результат, использование услуг

## Abstract

**Background.:**

The Finnish psychiatric treatment system has undergone a rapid transformation from operating in institutional settings to a adopting a community-based approach, through implementation of national plans; this process was carried out quickly, due to a severe economic recession in the early 1990s.

**Methods.:**

This paper is a narrative review, based on relevant documents by national authorities, academic dissertations and published scientific literature, between 1984 and 2018, as well as the interviews of key experts in 2019.

**Results.:**

The municipality is currently the primary organization, responsible for all health services. Municipalities may also work together in organizing the services, either through unions of municipalities or hospital districts. Services are to a great extent outpatient-oriented. The number of beds is one fifth of the previous number, around four decades ago, despite the increase in population. In 2017, 191,895 patients in total (4% of Finns) had used outpatient psychiatric services, and the number of visits totalled 2.25 million. Psychotherapy is mainly carried out in the private sector by licensed psychotherapists. Homelessness in relation to discharged psychiatric patients has not been in evidence in Finland and deinstitutionalization has not caused an increase in the mortality rate among individuals with severe mental disorders.

**Conclusion.:**

Psychiatric patients have, in general, benefitted greatly from the shift from institutions to the community. This does not preclude the fact that there are also shortcomings. The development of community care has, to date, focused too heavily on resource allocation, at the expense of strategic planning, and too little on methods of treatment.

## INTRODUCTION: FROM COMMUNITY TO INSTITUTIONS

Originally, individuals with severe mental disorders were cared for by relatives or by the church in Finland. During Swedish rule, there was no specific legislature regulating the “care of the delirious”. In 1840 during Russian rule, Czar Nikolai I issued a decree relating to the care of “mental diseases” and their treatment. The decree ruled that the state was the responsible organization of hospital care, and that several hospitals were to be built. The first hospital, established solely for the care of mental disorders, was opened in 1841 in Helsinki (Lapinlahti Mental Hospital). In 1880, a new decree, issued by Czar Alexander II, came into force, ruling that municipalities are responsible for the care of chronically mentally ill individuals, released from hospital. Specialist departments for the mentally ill were established thereafter in municipal homes, that provided housing and care for citizens, unable to care for themselves. These were functional until the establishment of “B-mental hospitals” (hospitals for chronically ill patients with psychoses) in the 1950s. The first outpatient office was founded by the Finnish Association for Mental Health in Helsinki in 1927 [1,2].

During Finnish independence, and since 1917, the first “law on mental illness” came into force in 1938. Municipalities were instructed to take more responsibility for the organization of psychiatric services, with economic support from the state. Several mental hospitals with a defined regional catchment area were built. According to the “law on mental illness”, close relatives or a legal guardian of a person, could apply for treatment in a mental hospital or a municipal home. Release from the hospital was often dependent on those, who had applied for the treatment. Commitment and use of involuntary measures were not yet explicitly regulated. A reform of the law on mental illness was issued in 1952 [1]. A comprehensive coverage of good services based on access to hospitals was a central aim for service development at the time. Mental departments in municipal homes were closed, and B-hospitals for the chronically ill were established around the country. In addition, the municipalities were obliged by law to establish “care offices” for patients, discharged from the hospitals. In 1977, a partial revision of the law on mental illness regulated the process of commitment and discharge in more detail than previously, making physician the sole responsible agent according to this act [2].

## MATERIAL AND METHODS

This paper is a narrative review, based on relevant documents by national authorities, academic dissertations and published scientific literature between 1984 and 2018, as well as information from key experts in 2019.

## FROM INSTITUTIONS TO COMMUNITY

At the end of the 1970s, before the deinstitutionalization process began in Finland, there were a total of 4.2 beds per 1,000 inhabitants in psychiatric hospitals, which at the time, were managed by 21 mental health districts. In Europe, only Ireland had a higher rate of beds [2]. Progress regarding outpatient-oriented care was initiated by the National Board of Health in 1978, and largely carried forward by mental health professionals from the early 1980s to the early 1990s. Resources for outpatient care were increased according to plans formulated in the 1980s, and the number of hospital beds decreased considerably around the turn of the decade. The number of hospital beds were reduced in greater numbers and over a shorter time period than originally planned, and the number of staff transferred from hospitals to outpatient care, was actually lower than originally anticipated. Until the 1980s, elderly patients with severe forms of dementia and individuals with mental retardation, were treated in psychiatric hospitals. These patient groups were transferred to other services during the deinstitutionalization process [2,3].

In 1991, a new Mental Health Law came into force. The principles of the new law were largely outlined by psychiatrists, working at the time in the National Medical Board. Outpatient services were defined as the front-line of care and included both health centres as well as psychiatric outpatient clinics. The health centres took care of patients with common mental disorders and assessed the needs of patients for specialized psychiatric care. In addition, compulsory care and the use of coercive measures, such as seclusion and restraints, were increasingly regulated. Subsequent revisions have included more detail in this regulation [4,5].

Additionally, two other important new laws came into force in the early 1990s [4,6]. A law for specialized healthcare integrated the previously separate mental health districts, responsible for psychiatric hospital treatment, with 21 healthcare districts. An additional law provided guidance in relation to the funding of public services, to compensate for the differences between the municipalities in demographic and economic conditions. This law strengthened the independence of the municipalities, and as a result, the health services in Finland became very decentralized. The regional variation of organization and the quality of the health services increased greatly [4,5].

### Services in the 21st century

Municipal social welfare and healthcare services, implemented with government support, form the basis of the social welfare and healthcare system. Private companies also provide services in addition to the public sector. Furthermore, Finland has a wide range of social welfare and healthcare organizations, providing services both free of charge and for a fee. The Ministry of Social Affairs and Health prepares legislature and steers its implementation. Policy guidelines are defined and reforms are prepared, guided and coordinated by the ministry. Agencies and institutes within the ministry oversee research and development (the Finnish Institute for Health and Welfare, THL and the Finnish Institute of Occupational Health), sanctioning medications (the Finnish Medicines Agency, Fimea) and radiation safety (Radiation and Nuclear Safety Authority, STUK).

Finland is divided into 21 hospital districts, which organize general hospital treatment and psychiatric hospital treatment for most of the municipalities. Finland comprises 310 municipalities, with a total population of circa 5.5 million. The number of inhabitants in a municipality varies from 690 to over 650,000, which has led to versatile administrative arrangements. Municipalities may organize primary healthcare and psychiatric outpatient care services independently, provided they have a sufficiently large population base to ensure fiscal sustainability. Municipalities may also work together to organize primary healthcare services and psychiatric outpatient care, either as a member of “unions of municipalities” or hospital districts. The districts are governed by representatives from the municipalities, and the districts receive their funding mainly from the municipalities. Municipal social services are responsible for home services, rehabilitative work activities and housing and community rehabilitation services, which, for the most part are currently run by private companies and non-governmental organizations (NGOs).

Primarily in the larger cities in Finland, hospital services have been organized by the cities themselves. Likewise, certain municipalities have also organized their outpatient care, whereas in other municipalities, outpatient care has been organized by hospital districts. Therefore, until the turn of the century, the organization and administration of the services have varied in a manner that renders a simple description cumbersome. Manpower varies greatly in psychiatric services, and there are currently no concerted data relating to this issue. There are roughly 1,800 physicians, specializing in one of the psychiatric disciplines. More than 1,000 of these specialists are of working age. By comparison with most European countries, Finland has a greater ratio of psychiatrists and psychiatric nurses per inhabitant. There are more than 5,600 psychologists in Finland, however, some of these work in areas other than mental health services. Around 4,000 of them work in services run by the public sector.

**Table 1:** Table 1. Levels of organization and corresponding responsibilities

Ministry of Social Affairs and Health	Health Districts	Municipalities	A-Clinics Ltd.	Private sector	NGO´s
+Legislature and its implementation +Policy guidelinesReforms of services +Agencies and Institutes: the Finnish Institute for Health and Welfare (THL), and the Finnish Institute of Occupational Health (ICH), the Finnish Medicines Agency (Fimea), Radiation and Nuclear Safety Authority (STUK) +Guidance and cooperation; prevention and promotion	Five separate university hospitals that comprise specific areas of responsibility +Hospital services: general and psychiatric, adolescent psychiatric and child psychiatric, (in certain districts) detoxification units +Outpatient psychiatric services, adolescent psychiatric and child psychiatric outpatient care +Outsourced psychotherapy services +Social services +Offices providing educational services or advice for families with under-age childrenHome services, rehabilitative work activities, housing and community rehabilitation services	Primary healthcare and social services +In larger cities: hospital services, general and psychiatric, adolescent and child psychiatric services +In larger cities: psychiatric, adolescent psychiatric and child psychiatric outpatient care +Outsourced psychotherapy services +Youth clinics +Prevention and promotion	Alcohol and substance abuse servicesAddiction hospitals +Detoxification units +Opiate dependence substitution treatment +Outpatient and digital services +Housing services +Family and youth services	General outpatient health services, operative hospital services +Psychiatrists’ surgeries +Psychotherapy servicesHousing services +Rehabilitation +Addiction services	MIELI: Mental health prevention and promotion +FINFAMI: promotion of mental health +Union of Mental Health: peer-support and other activities +Nyyti: student mental health promotion +Other smaller NGOs: mental health promotion, rehabilitation funded by SII

There is no longer specialized treatment for geriatric psychiatry, although patients older than 65 or 68, depending on the region, are treated in separate wards and outpatient units. Child psychiatry, adolescent psychiatry and general psychiatry are separate disciplines in Finland. Therefore, the treatment of children, adolescents and adults is carried out in separate units. The Mental Health Law stipulated that adolescents under the age of 18 are not to be treated in the same wards as adults. Outpatient care is also carried out in separate units. The upper age limits in psychiatric outpatient care, separating children, adolescents and adults vary between regions: children from 12 to 14 years, adolescents from 19 to 22 years and adults from 20 to 23 years [4,6,7]. Additionally, social services within the municipalities or unions of municipalities, have offices providing educational services or advice for families with under-age children.

If there is a reason why a suspect is not criminally responsible for a violent crime (manslaughter or homicide), the defence lawyer or the court may request an assessment of the psychiatric condition of a suspect. This assessment is conducted either in a state mental hospital, a psychiatric hospital for prisoners, or in a forensic department of a university hospital clinic, if such an institution exists. Should the perpetrator, suspected of having committed manslaughter or homicide, be deemed not to be criminally responsible, he/she will receive forensic psychiatric care in one of two state mental hospitals (Vanha Vaasa and Niuvanniemi). Additionally, psychotic patients with severe behavioural problems, who are difficult to treat and who are unable to be treated in the psychiatric hospitals themselves, are sent to state mental hospitals.

Psychotherapy is primarily carried out in the private sector by licensed psychotherapists, who have received a specialized education, lasting between four and six years. The key methods used are cognitive-behavioural, psychodynamic, solution-focused and trauma therapy. The Social Insurance Institution (SII) will reimburse around 60 to 80% of the fees of the psychotherapists – depending on how much they charge – for up to three years. Psychotherapy is funded by the SII as rehabilitation, to avoid disability or to promote a return to work-life. A statement by a psychiatrist is necessary to gain access to psychotherapy as rehabilitation. Currently, more than 50,000 individuals receive psychotherapy as rehabilitation annually (Metsä, personal communication). Access to psychotherapy tends to vary greatly within the country, as most therapists work in cities that have a university with a medical faculty. Psychotherapy services are also provided in the private sector in terms of outsourced psychotherapy, in larger cities and health districts.

NGOs provide mental health services free-of-charge and for a fee. Mental Health Finland (MIELI) has e.g., organized a national network of voluntary crisis counsellors, who work on telephone helplines. FINFAMI (Finnish Central Association of Families of People with mental illness) provides support with its member associations for families of people recovering from mental illness. Peer-support and activities are provided by the Mental Health Union, together with its member associations. Nyyti is an NGO which promotes the mental health of students in Finland.

### Addiction services

Treatment for psychiatric disorders and alcohol abuse has been carried out for decades in separate systems. Over the past three decades there has been an increasing effort to encourage integration. Currently, the primary care providers for individuals suffering from alcohol abuse are health centres and the A-Clinic Ltd., which is a non-governmental and non-profit organization, owned by the A-Clinic Foundation. A-Clinics offer a wide range of addiction services, such as outpatient therapy, detoxification units, housing services and hospital care in Järvenpää Addiction hospital. Health centres mostly screen patients with addictions, treat patients with milder forms of addiction and assess patients’ needs for specialized care, either within psychiatric services or within A-Clinics Ltd.

Addiction psychiatry units have been founded within psychiatric services. These units may take care of both hospital and outpatient care or solely the outpatient care of patients with illicit drug abuse. Previously, patients with alcohol delirium were treated in psychiatric hospitals, however, these patients are now treated in the detoxification units of general hospitals or, if necessary, in intensive care units. The primary responsibility assessment of opiate dependence is carried out by the addiction psychiatry units. If a patient is accepted into opiate substitute care, the treatment may be delegated to the A-Clinics or primary healthcare. Patients suffering from severe withdrawal states or psychoses due to drug abuse, are treated in psychiatric hospitals. For under-age patients with addictions, there are youth clinics, organized by municipalities or unions of municipalities. There are counsellors in schools, and education services may also provide psychological services to a certain extent.

### Costs of services

All public health and social services are primarily funded by municipal taxes and to some extent state taxes (state funding compensates the inequalities between municipalities and special state funding in terms of training and research for hospitals). The municipalities have always paid for the treatment, using funds from municipal taxation and to a lesser extent from state subsidies, irrespective of whether the services were organized by the municipalities themselves, by the unions of municipalities, by the hospital districts or by state mental hospitals. Health districts and state mental hospitals bill the municipalities for the number of patients treated (taking into account the number of visits and days in hospitals). If the municipalities cannot afford to pay for all treatment given, the hospital districts cover the missing portion of the payments. Mental healthcare costs are circa 9% of all healthcare costs in Finland.

Patients using the private sector can be reimbursed to a certain degree by the SII [4,5], which constitutes funded state taxes. Patients may also receive reimbursements for rehabilitative psychotherapy and medications, prescribed by a physician. Psychiatric outpatient care visits are free-for-charge, with the exception of an appointment not being cancelled on time. The only out-of-pocket expense for a patient in public outpatient care, is payment for approximately half the medication required. Hospital care is to a lesser extent covered by the patients themselves, and if they cannot afford the payments, social services will lend a helping hand.

## NEW DEVELOPMENTS

A major, current trend regarding both psychiatric outpatient and hospital services, has been a move towards a larger population base. Hospital and specialized psychiatric outpatient care, previously organized by certain municipalities, has been or is being integrated into hospital districts. The government of Finland has outlined a plan to shift the responsibilities of health and social services from the municipalities to larger areas or provinces, which would reduce the diffuse ways of arranging and administrating healthcare. Social and healthcare services would also be more integrated as a result, than they are currently.

Around a decade ago, the authorities took the decision that the old mental hospitals, treating psychiatric patients exclusively, would be closed, and psychiatric hospital treatment would be integrated with general hospitals. There were two key motivations for this undertaking. Firstly, the quality assurance of the patient’s treatment as a whole, was considered of utmost importance. Psychiatric patients have a higher rate of mortality, due to somatic illnesses and they do not receive as much care as the general population for their illnesses [8]. For instance, imaging and laboratory services were not available in separate mental hospitals, and intensive care units are at times necessary e.g., for patients in severe delirious states. Secondly, an attempt to decrease stigma was also an important factor. Psychiatric patients would not in the future be segregated from other patients, although they would continue to have their own department. Many new hospital units are, therefore, being built today in Finland, but at the same time, there will be a further decrease in the number of beds.

During the past decade, service development has been outlined in two mental health plans, Mind2009 and Mind2015 [9]. Presently, a new plan is being drafted. One main aim of Mind2009 is to integrate addiction and psychiatric services. Although cooperation between the services provided for these patient groups is improving, and the quality of treatment is of a higher standard, there are still shortcomings in relation to their integration. This is largely due to the fact that addiction services have been operating separately from other mental health services for decades. Mind2015 focuses on emphasizing patient-centredness, the promotion of mental health and abstinence, the integration of somatic and psychiatric care, the integration of administration and the development of a means for measurement-based administration.

A new innovation, that of a model of triadic cooperation, was developed for occupational health services and health centres. In this treatment model, nurses are trained to provide either group or individual psychotherapeutic treatment for patients [10]. An occupational healthcare physician or general practitioner takes care of prescribing medication, and a psychiatrist is available for consultation, if the treatment does not proceed as planned. The method has not been in use throughout the country, however, Etelä-Pohjanmaa hospital district has been able to close a ward due to a decrease in demand for psychiatric hospital care (Lassila personal communication).

## USE OF SERVICES IN 2017

In 2017, there were 195,406 patients within specialized psychiatric services, including both outpatient and inpatient care. The number of patients who received hospital care was 24,495 and the number of treatment episodes was 37,705. Within the past decade there has been a slight decrease in the number of patients. The number of patients treated in psychiatric hospitals has decreased greatly during the past three decades, and treatment episodes are considerably shorter. Even within a decade, from 2006 to 2017, the rate decreased by more than 20% [11]. More than half of the patients have hospital treatment episodes that last less than two weeks, while less than 1% receive hospital treatment for more than a year. The latter group primarily comprises patients in state mental hospitals, who benefit from forensic psychiatric services and who constitute difficult-to-treat patients.

The number of outpatients has increased within the past decade by ca. 65,000. In 2017, 191,895 patients in total had used outpatient psychiatric services, and the number of visits was 2.25 million [11]. On the other hand, these numbers do not include visits to private psychiatric or psychotherapeutic services. Around a fifth of working age psychiatrists work in the private sector. In recent years, the number of referrals to psychiatric outpatient care and the use of psychiatric services, has increased without any reliable indication of a coinciding increase in the incidence of mental disorders. The stigma due to mental disorders has decreased dramatically within the past few decades. Another important factor may be that many health centres have lacked qualified general practitioners, thus limiting the access to primary healthcare on time.

There is a large variation in the use of services in Finland, and the prevalence of use does not follow the prevalence of disorders. Variation in terms of access to services is one key factor, which is dependent on the availability of adequate services. The rate of treatment episodes is relatively evenly distributed but the length of hospital stays per episode, varies greatly. Previously, in the 1980s and 1990s, the length of stays correlated with the availability of outpatient services [12,13].

During the 1990s, due to increasing decentralization, the development of treatment and rehabilitation methods, and the settings for individuals with severe mental disorders began to vary. Most housing services are run by private companies or NGOs, some of which also provide adequate rehabilitation services. The quality of supported housing and rehabilitation to be carried out in these units, currently varies greatly. There are more than 7,000 people with severe mental disorders who live in these units, and the level of service they receive may sometimes be of a lower quality than the service provided some decades ago in the hospitals for chronically ill psychiatric patients (Kärkkäinen, personal communication).

In a recent study, the European Service Mapping Schedule-Revised (ESMS-R) tool was used to classify the adult mental health service (MHS) structure in southern Finland (population 1.8 million, 18+ years).13 The diversity, including various types of day-care and outpatient services, of the MHS was found not to be associated with hospitalization. Only a general index of mental health needs was associated with an increased use of inpatient treatment. The researchers concluded that strategic planning is quintessential in service-planning and that an increase in the number of resources in outpatient services, is not sufficient to decrease the need for hospital care, as inpatient care is associated with factors relating to population and the healthcare system. In the same research project, it was also found that the diversity of services is dependent on the size of the population base. A minimum of 150,000 inhabitants are needed to justify a diverse mental healthcare system and to satisfy the multiple needs of psychiatric patients [14].

## CERTAIN VIEWPOINTS RELATING TO OUTCOME

A suicide prevention project was carried out in Finland between 1987 and 1996. Firstly, psychological autopsy studies were conducted. The studies showed that two thirds of individuals who committed suicide, suffered from a clinically significant depressive illness that was often under-treated. After the study phase, an intervention was planned, implemented and subsequently, evaluated. During this project physicians received training on the identification and treatment of depression [15]. At the same time, new antidepressants became available and their use increased greatly even until the 2010s. During the 1990s, the rate of suicides decreased dramatically, and one factor seems to be the increased use of antidepressants among the male population, who previously had not received adequate treatment for their condition [16].

Homelessness in relation to discharged psychiatric patients has not been in evidence in Finland and deinstitutionalization has not increased mortality among individuals with severe mental disorders. In fact, a seminal study showed that in those regions of Finland, where the coverage of outpatient services was high, the rate of suicide was lower than in regions where the treatment system was more hospital-oriented [17]. However, there is an excess mortality among patients with severe mental disorders. In psychoses, excess mortality was found to be 3.5-fold and in psychoactive substance abuse, 5.3-fold, by comparison with the general population. Overall, the mortality of the population and patients with severe mental disorders has decreased between 1996 and 2010.

In 2000, in a population-based sample, 31% of those who had experienced a major depression episode, received pharmacological, psychotherapeutic or both types of treatment. Slightly less than a fifth of those suffering from depression received treatment that was barely adequate. However, only a third of individuals with major depression, made use of the health services for mental healthcare. The treatment coverage was considerably better among the service users, as 76% received antidepressants, psychotherapy or both. Under-treatment is thus primarily a problem of objective needs, not resulting in the use of services. The majority of those using health services for mental reasons were of the opinion that the care had been quite or very helpful. The level of satisfaction was even higher among those who had received psychotherapy. In the same sample, most (80%) of those with anxiety disorders, who used health services for mental health reasons, received pharmacotherapy, however, less than half received any form of psychosocial or psychotherapeutic treatment [18,19].

Among the employees of 10 Finnish municipalities, psychotherapy, funded as a means of rehabilitation was considered an effective form of treatment. Patients with major depression, who had long absence periods from work due to mental health issues (>21 days) before psychotherapy or antidepressant treatment, reported a significant decrease in the ratio of sickness absence at the end of the entire follow-up, compared with absence from work before or during the treatment. During the follow-up, healthy controls noted an increase in sickness absence. Psychotherapy and antidepressant treatment were associated with a substantial decrease in sickness absence for at least six years after the end of treatment [20].

All outpatients were routinely asked to fill in questionnaires on symptoms and their health-related quality of life at the baseline and after three, 12 and 24 months in a research and development project on outpatient psychiatric care, at Satakunta Hospital District in Finland between 2010 and 2014. The project found that for most patients, recovery was highly clinically significant and was defined as a change in the health-related quality of life. Recovery after one- and two-year follow-ups depended to a large extent on the recovery at three months. This result emphasizes the importance of measurement-based psychiatry. Quality of life could be a useful generic outcome measure. Following the patients´ state routinely by an outcome measure, could provide benefits for the treatment of those patients, in particular, who do not recover at all or who recover slowly during the first three months.

## DRAWBACKS OF THE SERVICES

Schizophrenia patients who are difficult to treat are increasingly referred to state mental hospitals, which are the only psychiatric hospitals, in which the number of wards and beds have increased. A similar development has been found in Denmark [21]. Moreover, access to the rehabilitation of individuals with severe mental disorders may be somewhat arbitrary, as the quality of services in supported housing seems to vary greatly. If the governing principle is simply inexpensiveness, then the companies have an incentive to retain the residents and not rehabilitate them to achieve independence. However, currently, there are improving trends in relation to rehabilitation and housing services.

Outpatient care in community clinics seems to lack intensity and continues over too long a period of time, resulting in new patients not having timely access to intensive treatment. Less than a fourth of patients in community clinics receive visits on a weekly basis, over a two- to six-month period. In the case of 75%, the mean frequency of visits is below 0.6/week. Yet, this care, based on infrequent visits, may continue for a number of years. A large proportion, even around 50% of the input staff in outpatient clinics, is focused on treating patients, who have been receiving treatment for several years (Horjamo personal communication). Additionally, outpatient services, generally, do not provide evidence-based, time-limited, psychotherapeutic treatment for patients in the acute phase. To date, the only choice has been rehabilitation psychotherapy, funded by the SII. There is an increasing consensus that evidence-based psychotherapy should be included as a key component of outpatient care in future development plans.

The health benefits of a large proportion of the visits to outpatient clinics may, thus, be questionable, due to the lack of intensity and specified treatment plans. Therefore, new patients may not receive care that is adequately intensive and comprehensive, which again may result in shortcomings in terms of recovery, sickness absence or disability. The effectiveness of the treatment and the feedback from patients are not measured routinely. Should such monitoring not be carried out, it is very difficult or perhaps impossible to understand which needs must be addressed. Cost-effectiveness is, likewise, not followed-up, which hinders the development of a fiscally sustainable plan.

## CONCLUSION

Psychiatric patients have, in general, benefitted greatly from the shift from institutions to the community. This does not preclude the fact that there are also shortcomings. The development of community care has, to date, focused too much on resource allocation at the expense of strategic planning, and too little on the type of treatment. Furthermore, since attention has been focused on shifting resources from hospitals to outpatient care, there has not been a similar development of treatment, carried out in the hospitals.

## Conflicts of interest

The author declares no conflict of interest.

## Funding

The article was written without external funding.
